# Antileukemic Activity and Molecular Docking Study of a Polyphenolic Extract from Coriander Seeds

**DOI:** 10.3390/ph14080770

**Published:** 2021-08-05

**Authors:** Hamza Mechchate, Regiane Costa de Oliveira, Imane Es-safi, Emmily Myrella Vasconcelos Mourão, Mohamed Bouhrim, Andrii Kyrylchuk, Gemilson Soares Pontes, Dalila Bousta, Andriy Grafov

**Affiliations:** 1 Laboratory of Biotechnology, Environment, Agri-Food, and Health (LBEAS), Faculty of Sciences, University Sidi Mohamed Ben Abdellah (USMBA), Fez B.P. 1796, Morocco; Imane.essafi1@usmba.ac.ma (I.E.-s.); dalila.bousta@usmba.ac.ma (D.B.); 2 Post-Graduate Program in Haematology, School of Health Sciences, University of the State of Amazonas, Av. Djalma Batista 3578, Manaus 69050-010, AM, Brazil; regiane.costa17@gmail.com (R.C.d.O.); pontesbm1@gmail.com (G.S.P.); 3 Laboratory of Virology, National Institute of Amazonian Research (INPA), Av. André Araújo 2.936, Petrópolis, Manaus 69067-375, AM, Brazil; vasconcelosemmily@gmail.com; 4 Laboratory of Bioresources, Biotechnology, Ethnopharmacology and Health, Faculty of Sciences, Mohammed First University, Oujda B.P. 717, Morocco; mohamed.bouhrim@gmail.com; 5 Institute of Organic Chemistry, National Academy of Sciences, Murmanska Str. 5, 02660 Kyiv, Ukraine; iamkaant@gmail.com; 6 Department of Chemistry, University of Helsinki, A.I. Virtasen aukio 1, 00560 Helsinki, Finland

**Keywords:** leukemia, *Coriandrum sativum* L., polyphenols, HL60, K562, Vero cell line, anticancer activity, molecular docking, *ABL* kinase, *ABL1*, *BCL2*, *FLT3*, acute toxicity, OECD 423

## Abstract

Leukemia is a group of hematological neoplastic disorders linked to high mortality rates worldwide, but increasing resistance has led to the therapeutic failure of conventional chemotherapy. This study aimed to evaluate in vitro the antileukemic activity and potential mechanism of action of a polyphenolic extract obtained from the seeds of *Coriandrum sativum* L. (CSP). A methylthiazoletetrazolium assay was performed to assess the CSP cytotoxicity on chronic (K562) and acute (HL60) myeloid leukemia cell lines and on normal Vero cell line. CSP toxicity was also evaluated in vivo using the OECD 423 acute toxicity model on Swiss albino mice. The results demonstrated a remarkable antitumoral activity against K562 and HL60 cell lines (IC_50_ = 16.86 µM and 11.75 µM, respectively) although no cytotoxicity was observed for the Vero cells or mice. A silico study was performed on the following receptors that are highly implicated in the development of leukemia: *ABL* kinase, *ABL1*, *BCL2*, and *FLT3*. The molecular docking demonstrated a high affinity interaction between the principal CSP components and the receptors. Our findings demonstrated that CSP extract has remarkable antileukemic activity, which is mainly mediated by the flavonoids, catechins, and rutin, all of which showed the highest binding affinity for the targeted receptors. This study revealed a promising active compound alternative research-oriented biopharmacists to explore.

## 1. Introduction

Leukemia is a heterogeneous group of hematological diseases characterized by the uncontrolled and dysfunctional growth of leukocytes [1]. Leukemia and nervous system cancers represent the primary causes of cancer mortality in men under the age of 40 and women under the age of 20 [2]. In 2020, approximately 60,530 new cases of leukemia were diagnosed, and 23,100 people are expected to die from this malignancy [2].

Human leukemia is caused by a combination of mutations that result in defects of gene expression, disrupting the fragile equilibrium of replication, differentiation, and apoptosis [3]. Cloning translocation breakpoints have given important insights into the pathogenesis of this disorder. Mutations in transcription factor genes involved in normal hematopoietic production are among the chromosomal aberrations seen in acute myeloid leukemia [4]. Mutations affecting transcription factors and key cell genes result in uncontrolled proliferation and compromised differentiation [5]. The understanding of the genes involved in the pathogenesis of leukemia has opened the way for novel therapeutic strategies that target different gene products implicated in cancer progression. Targeted treatments for hematological malignancies have climbed to the top of the list of leukemia care choices [6]. The most well-known therapies are all-trans retinoic acid (ATRA) for treating acute promyelocytic leukemia (APL) and imatinib mesylate (Gleevec) that targets a BCR-ABL oncogene in chronic myeloid leukemia (CML) [6,7].

Chemotherapy is still the major therapeutic strategy in leukemia treatment [8]. However, the available drugs possess a high and unspecific cytotoxicity that severely affects normal cells as well. Moreover, the increasing resistance of cancer cells to treatment remains a significant obstacle to successful chemotherapy [9]. Therefore, looking for potential anticancer drugs from medicinal plants has steadily increased over the years [10].

Plants are an inexhaustible source of different classes of biologically active compounds [11]. Among those are polyphenols, which possess anticancer, antimicrobial, antiviral, anti-inflammatory, and tumor suppression properties and are cytotoxic [12,13].

Coriander (*Coriandrum sativum* L.; Apiaceae) is an edible plant native to a wide area of Eurasia and Northern Africa, where it is mainly cultivated for fresh leaves and dried seeds [14]. The latter is a common spice that has a plethora of documented traditional medicinal uses for treating diabetes, hypertension, indigestion, bloating, pain, rheumatism, renal disorders, and worms [14]. Earlier, some anticancer properties of coriander were reported. For example, the terpenoid linalool (a coriander seed oil component) exhibited a dose-dependent cytotoxic effect in vitro and led to a decrease in tumor volume and weight in vivo at the dose of 200 mg/kg bodyweight [15]. However, another in vitro study suggested that coriander leaves effectively block skin tumor initiation but fail to suppress the tumor promotion phase [16].

In a recent paper [17], we demonstrated that the polyphenol-rich fraction of the coriander seed extract contains several catechins, polyphenolic acids, and rutin.

Catechins, or flavan-3-ols, are present in a wide range of plants and dietary sources, such as wine, green tea, and cocoa. They are powerful antioxidants, though they may also serve as pro-oxidants throughout the cell [18]. They are ROS scavengers and metal ion chelators, and their indirect antioxidant functions include inducing antioxidant enzymes, inhibiting pro-oxidant enzymes, and producing phase II detoxification and antioxidant enzymes [19]. Catechins can be useful in avoiding and defending against diseases induced by oxidative stress because of their antioxidant properties [18]. 

Many attempts have been made in recent decades to learn more about the mode of action of catechins, especially in cancer treatment. For example, green tea consumption has been related to a mild reduction in the prevalence of cancers such as colorectal, lung, esophageal, and prostate [20,21]. Green tea intake has also been linked to a lower incidence of breast, ovarian, and thyroid cancer [22,23]. It is now apparent that the anticancer activity of catechins is no longer restricted to their direct antioxidant/pro-oxidant properties. They also may target lipid rafts and the endoplasmic reticulum, modulate gene expression by direct action on transcription factors or indirect epigenetic pathways, and interfere with intracellular proteostasis at various stages [24]. Phenolic acids are one of the important classes of bioactive chemicals grouped under the polyphenol family present in various plant sources such as fruit, vegetables, spices, grains, and beverages. They are aromatics that add color, taste, astringency, and harshness to the normal organoleptic properties of food [25]. Phenolic acids present a wide spectrum of anti-inflammatory, antioxidant, cardioprotective, antidiabetic, and anticancer health benefits [26,27]. For example, chlorogenic acid is recognized both for its nutritional and health benefits. Its activity against human breast, lung, colon, bone, and kidney cancers was demonstrated in vitro [28], while an in vivo study found antitumoral activity against colon cancer in rats [29]. Vanillic acid inhibited cell proliferation through a G1 phase arrest and an angiogenesis suppression, the antitumor activity was confirmed in a murine xenograft model with no apparent toxicity to the animals [30].

Rutin (quercetin-3-*O*-rutinoside) is a glycoside composed of the flavonol quercetin and a disaccharide rutinose found in asparagus, buckwheat, apricots, apples, cherries, tomatoes, grapefruit, plums, bananas, and tea [31]. It has a wide range of biological activity: antioxidant, anti-inflammatory, antiangiogenic, pro-apoptotic, and antiproliferative [11,32]. Its activity was demonstrated in a variety of cell models: adenocarcinoma, glioblastoma, leukemia, and cancer (breast, prostate, lung, stomach, liver, and colon) [33]. Rutin can inhibit cancer initiation and development through a variety of mechanisms, including modulating different dysregulated signaling pathways involved in inflammation, apoptosis, autophagy, and angiogenesis [31].

In this study, we evaluated the anticancer potential of a polyphenolic extract from *C. sativum* seeds against two leukemia cell lines and a molecular docking study was performed to reveal the potential mechanism of action of the extract through receptor-ligand analysis.

## 2. Results and Discussion

### 2.1. Extract Analysis

**Figure 1:**
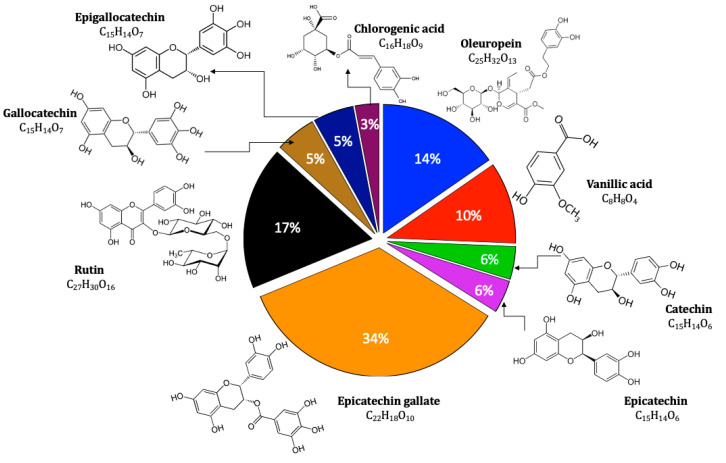
Components identified in *Coriandrum sativum* L. polyphenolic extract (CSP).

The polyphenolic extract of *C. sativum* seeds was prepared according to the method we proposed [17]. The LC/MS–MS revealed the presence of nine components in the prepared extract: vanillic acid, chlorogenic acid, catechin, epicatechin, epicatechin gallate, gallocatechin, epigallocatechin, oleuropein, and rutin. The content of those polyphenolic substances in the extract is shown in the Figure 1. The data relative to the analysis, which were determined by calculating the area under the curve generated by the fragments compared to that of the standards and blanks, are provided in the Supplementary Materials. The powerful antioxidant catechins (catechin, epicatechin, epicatechin gallate, gallocatechin, epigallocatechin) were the most represented [34]. Vanillic and chlorogenic acid are phenolic acids; oleuropein is a secoiridoid; and rutin is a flavonol glycoside.

Previous studies in animal models demonstrated that several of the above compounds have a wide range of cancer-preventive and chemotherapeutic properties, and various pathways are likely to be involved with a perfect synergy [35].

### 2.2. Antileukemic Activity

The cytotoxic activity of *C. sativum* polyphenolic extract was evaluated using three cell lines: human acute promyelocytic leukemia (HL60), human chronic myelogenous leukemia (K562), and normal Vero cell. The cytotoxicity indices were estimated as a cell viability percentage measured by a methylthiazoletetrazolium (MTT) assay in a dose-dependent manner after 24, 48, and 72 h of treatment with increasing doses (0 to 100 µg/mL) of the CSP extract.

**Figure 2:**
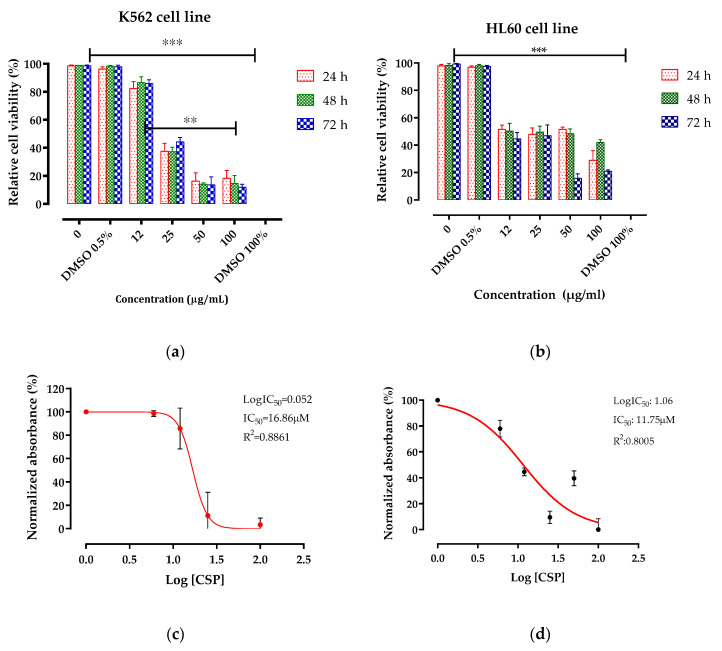
Cytotoxicity of the CSP extract for K562 and HL60 cells. (**a**) K562 and (**b**) HL60 cell viability after 24–72 h of treatment with different concentrations of the extract (12–100 µg/mL). The IC50 for K562 (**c**) and HL-60 (**d**) was estimated using nonlinear regression (GraphPad Prism v. 5 software). The absorbance values were measured at the wavelength of 570 nm and the mean values ± SD of three experiments are displayed along with a representative IC50 curve. The cell viability was estimated by the MTT assay. ** *p* < 0.01;*** *p* < 0.001.

**Figure 3:**
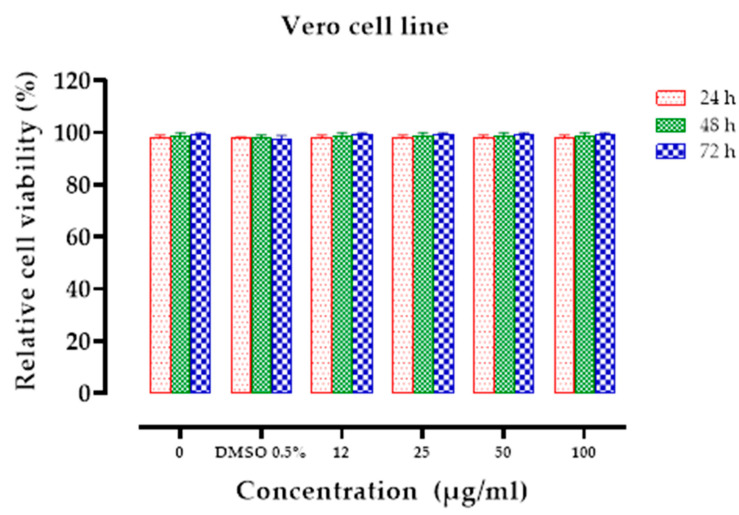
Cytotoxicity of the CSP extract for normal Vero cell line. Relative viability of Vero cells after 24–72 h of treatment with different concentrations of the extract (12–100 µg/mL). The absorbance values were measured at the wavelength of 570 nm and the mean values ± SD of three experiments, the cell viability was estimated by the MTT assay.

As shown in Table 1, the extract was able to inhibit the proliferation of HL60 (IC_50_ = 11.75 µM) and, K562 (IC_50_ = 16.86 µM) cancerous cell lines. The reduction in 50% of viable cell numbers was evident after a 24 h treatment period at a dose of 25 µg/mL for the K562 cell line and 12 µg/mL for HL60. A maximum cytotoxicity of 80% was reached at 50 µg/mL concentration after 24 h and 72 h for the K562 and HL60 cell lines, respectively (Figure 2). No cytotoxicity was observed on Vero cells at any concentration tested (IC_50_ > 100 µM) (Figure 3). Our findings demonstrated powerful activity and a selective cytotoxicity of the CSP extract that specifically targeted tumor cells (HL60 and K652) without affecting the normal ones (Vero).

The results obtained for the CSP extract suggest a potential synergistic effect between different components when compared with literature data for individual constituents (Table 1). 

### 2.3. Acute Toxicity Study

#### 2.3.1. Behavioral Studies

In the 14 days following the CSP extract treatment, no deaths or signs of toxicity were observed in the experimental animals. Accordingly, the approximate LD_50_ dose of the CSP extract in female mice was greater than 2000 mg/kg bodyweight.

#### 2.3.2. Relative Weight

**Figure 4:**
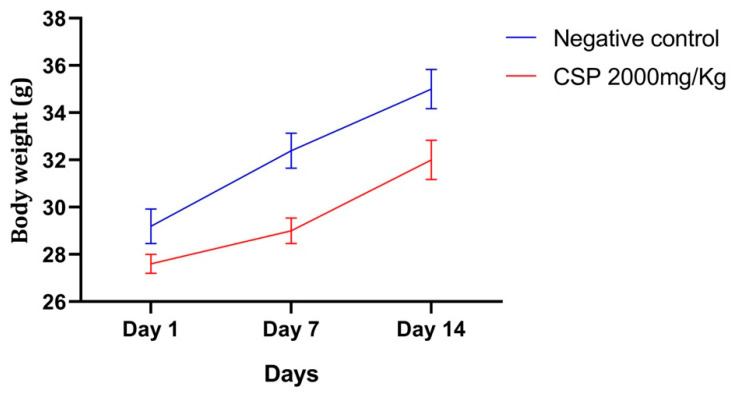
CSP effect on body weight changes in mice. Data presented as mean ± SD (*n* = 6).

Single oral administration of the CSP extract at a dose of 2000 mg/kg bodyweight did not interfere with the mice growth (Figure 4) and does not produce any effect on the general state of the mice during the 14 days, according to the OECD. Thus, oral administration of CSP is not toxic in a single dose. 

Neither abnormal behavioral changes nor the death of the treated animals was observed. The female mice treated with CSP extract at a dose of 2000 mg/kg had no symptoms of poisoning, respiratory failure, prolonged salivation, or diarrhea. 

Alterations in body weight and internal organs, which serve as early indicators of drug toxicity [44], but the female mice in both research groups gained weight gradually and were active during the whole study period, indicating regular food and water intake. Furthermore, there were no substantial differences in relative organ weights between the control and CSP-treated groups, indicating that the extract was not harmful to the internal organs (Table 2).

#### 2.3.3. Biochemical and Hematological Analyses

The variations in hematological and biochemical parameters of mice treated with CSP are listed in the Table 3 and Table 4 respectively. The study did not reveal any difference between the control and CSP-treated groups.

The hematopoietic system is a critical target for toxic agents, and thus is an important indicator of the physiological and pathological status of humans and animals [45]. There were no major variations between the treated and control groups in hematological analyses, showing that the CSP extract had no harmful effects on the hematopoietic system.

This study also assessed the lipid profiles and serum biochemical markers of renal and liver functions. No significant difference was observed in the serum levels of cholesterol, VLDL, HDL, or triglyceride between the control and treated groups. Likewise, the renal function was not affected by the CSP extract since both control and treated groups showed similar serum levels of urea and creatinine. The liver, known as the body’s biochemical center, is essential for maintaining metabolic homeostasis. Hepatic dysfunction disrupts the metabolic activities across the liver, increasing serum levels of biochemical markers like SGOT, SGPT, ALP, and bilirubin. No major differences in liver serum biochemical markers were found in the CSP-treated mice, indicating a total safety of the extract.

Side effects remain one of the biggest drawbacks of the acute myeloid leukemia treatment because chemotherapy has a strong impact on patients’ quality of life and can sometimes discourage the patient from continuing treatment [46]. Similarly with chronic myeloid leukemia treatment, the prescribed tyrosine kinase inhibitor imatinib and others are associated with cases of liver toxicity [47], chronic fatigue [48], nausea, rash, superficial edema, muscle cramps, and myelosuppression [49].

Finding an effective treatment with fewer or almost no side effects is the ultimate goal [50], and the present study was conducted from this perspective. Our results demonstrated the practical absence of CSP extract toxicity in vitro and in vivo, drawing more attention to its antileukemic activity and the need for further investigation into its mode of action and limitations.

### 2.4. Molecular Docking

Four receptors were selected as targets of high interest in antileukemic research and therapy (*ABL* kinase, *ABL1*, *BCL2*, and *FLT3*) [51,52,53,54,55,56]. The ligand-receptor affinity and the presence of interactions among them were chosen as criteria for a ligand–pose selection.

**Figure 5:**
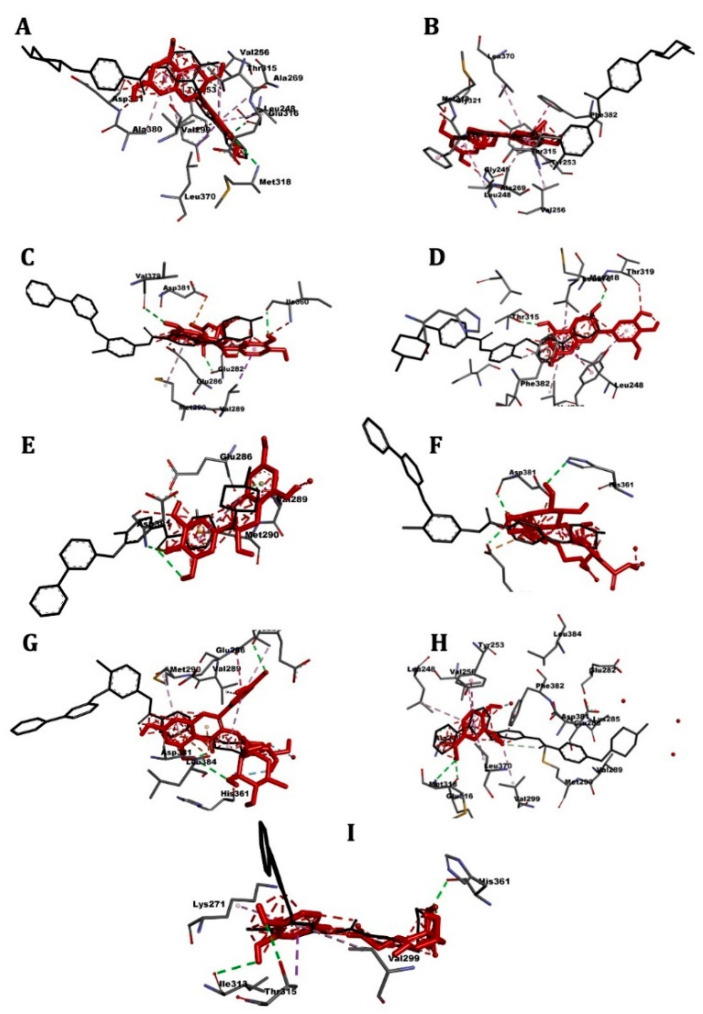
3D scheme of the ligand-ABL kinase receptor interactions. In red, tested molecule (**A**) catechin, (**B**) epicatechin, (**C**) epicatechin gallate, (**D**) epigallocatechin, (**E**) gallocatechin, (**F**) oleuropein, (**G**) rutin, (**H**) vanillic acid, (**I**) chlorogenic acid; in black, reference molecule (nilotinib) and labeled amino acid residues interacting with the tested molecule.

**Figure 6:**
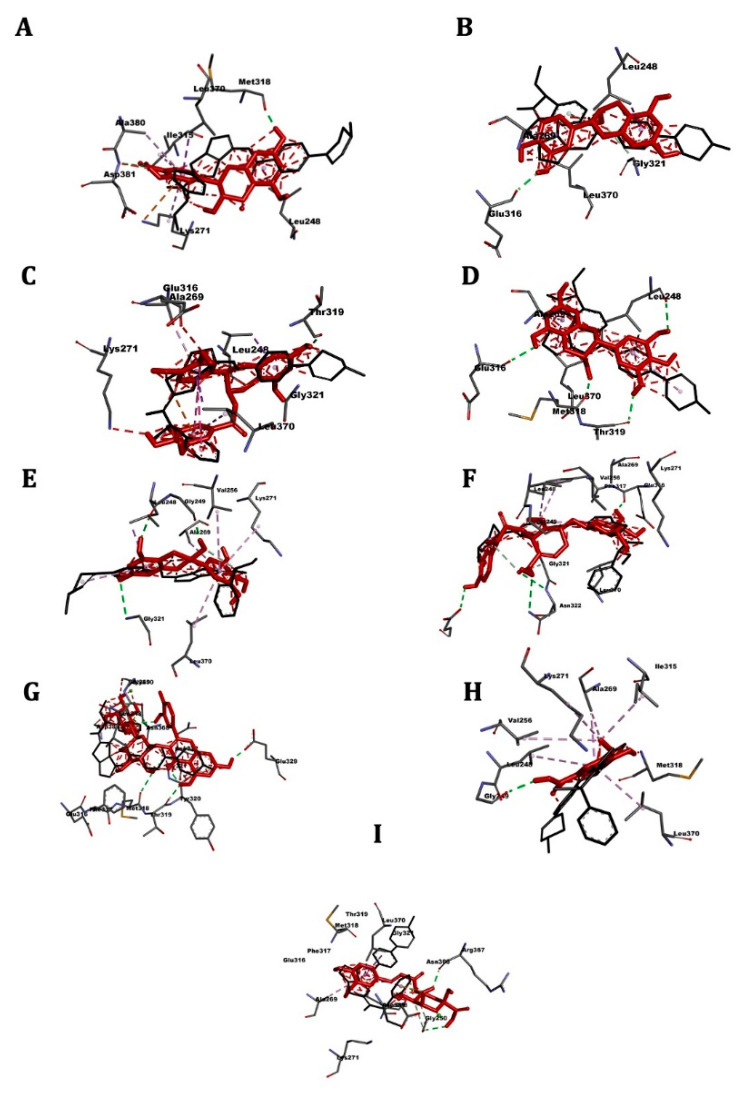
3D scheme of the ligand–ABL1 receptor interactions. In red, tested molecule (**A**) catechin, (**B**) epicatechin, (**C**) epicatechin gallate, (**D**) epigallocatechin, (**E**) gallocatechin, (**F**) oleuropein, (**G**) rutin, (**H**) vanillic acid, (**I**) chlorogenic acid; in black, reference molecule (danusertib) and labeled amino acid residues interacting with the tested molecule.

**Figure 7:**
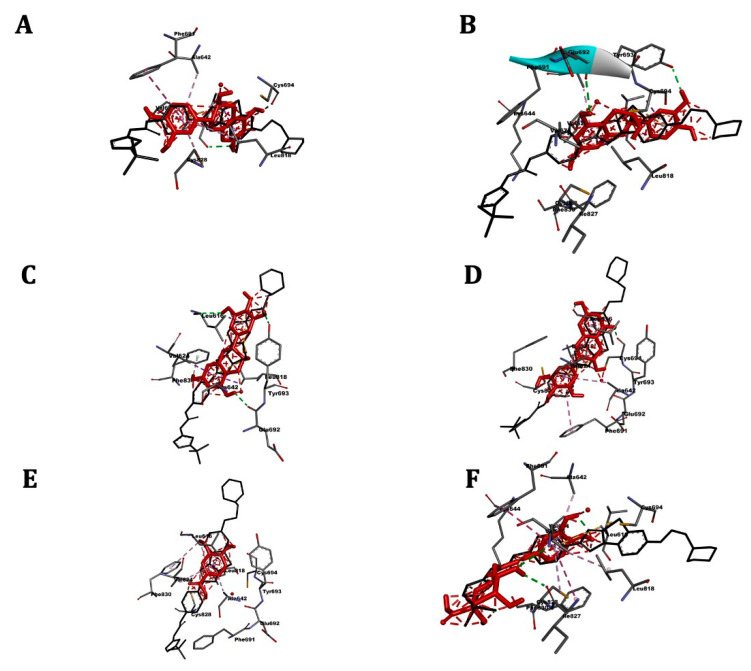
3D scheme of the ligand–FLT3 receptor interactions. In red, tested molecule (**A**) catechin, (**B**) epicatechin, (**C**) epigallocatechin, (**D**) gallocatechin, (**E**) vanillic acid, (**F**) chlorogenic acid; in black, reference molecule (quizartinib) and labeled amino acid residues interacting with the tested molecule.

**Figure 8:**
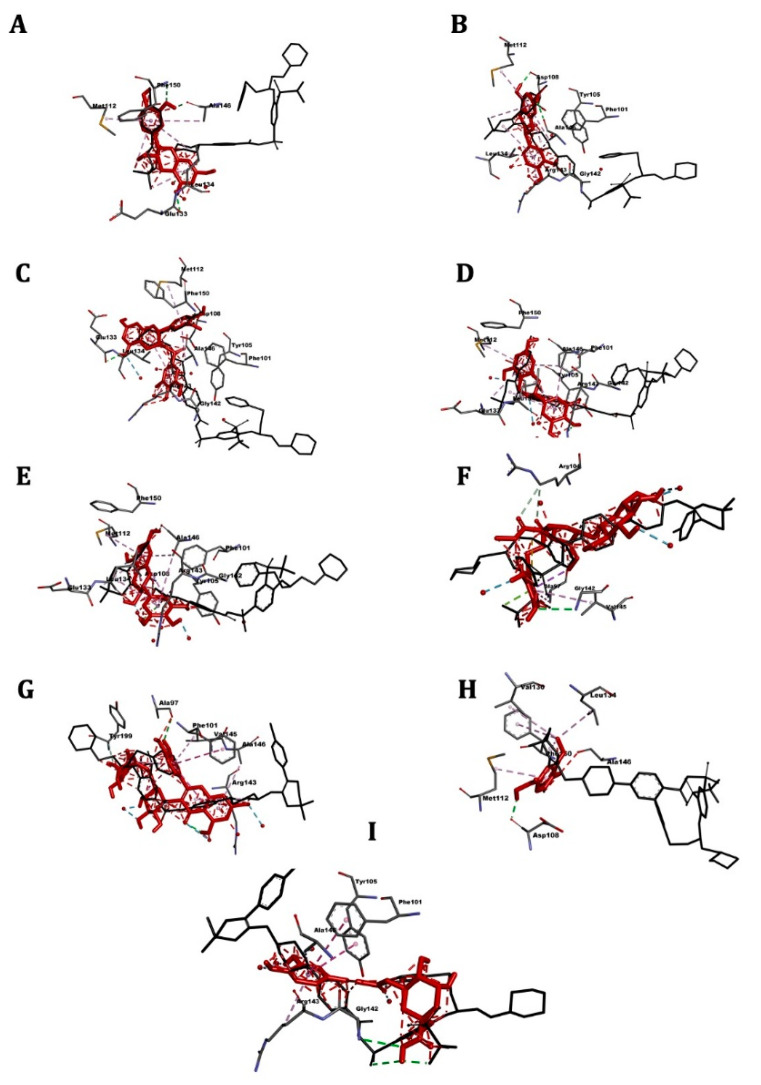
3D scheme of the ligand–Bcl-2 receptor interactions. In red, tested molecule (**A**) Catechin, (**B**) Epicatechin, (**C**) epicatechin gallate, (**D**) epigallocatechin, (**E**) gallocatechin, (**F**) oleuropein,(**G**) rutin, (**H**) vanillic acid, (**I**) chlorogenic acid; in black, reference molecule (navitoclax) and labeled amino acid residues interacting with the tested molecule.

Table 5 shows the affinity results and 3D images of different docking poses of the ligands with the receptors are provided in separated figures (Figure 5, Figure 6, Figure 7 and Figure 8).

### 2.5. ABL Kinase

The role of this protein is related to the development of the Philadelphia chromosome by the union of the *ABL* and *BCR* oncogenes, which is responsible for the phosphorylation and eventual proliferation of the oncogenic cells in chronic myeloid leukemia [51].

Molecular-docking results are present in Table 5 and Figure 5. The reference drug nilotinib showed the highest affinity for the receptor (−9.9 kcal/mol), the docking affinities of the CSP extract components ranged between −9.6 kcal/mol (epicatechin) to −6.5 kcal/mol (vanillic acid). The most active compounds from the extract were the catechins and rutin: −9.3 kcal/mol.

### 2.6. ABL1

Known as T315I, this mutant protein increases resistance to tyrosine kinase inhibitors. The major distinction between the T315I and *ABL* kinase proteins is the mutation of threonine to isoleucine at amino acid residue 315 (T315I), which weakens the association of inhibitors with those residues. Thr315 is responsible for the mechanism of inhibition of protein BCR–ABL [52].

Molecular docking results are present in Table 5 and Figure 6. The reference drug danusertib showed an affinity for the receptor of −8.5 kcal/mol. The best affinity was found for the epicatechin gallate −9.2 kcal/mol, followed by rutin (−8.6 kcal/mol). Vanillic acid was the least active (−5.4 kcal/mol).

### 2.7. FLT3

*FLT3* is a protein of the tyrosine kinase receptor family that is mainly involved in stem cell proliferation and differentiation [53]. Wildtype *FLT3* is expressed in a variety of hematopoietic malignancies, including acute lymphoid leukemia (AML), mixed lineage leukemia, and, most prominently, in acute myelogenous leukemia [54].

Molecular docking results are present in the Table 5 and Figure 7. The reference drug quizartinib showed the highest affinity for the receptor (−10.1 kcal/mol). The docking affinities of the CSP extract components ranged between −9.1 kcal/mol (catechin) to −5.7 kcal/mol (vanillic acid). Catechins were the most active compounds of the extract.

### 2.8. Bcl-2

In natural hematopoiesis, the balance of replication, apoptosis, and differentiation is achieved by the cooperation of pro-and anti-apoptotic genes. In cancer cells, this delicate equilibrium is compromised, and a number of mutations enhance anti-apoptotic gene expression, tipping the scales in favor of proliferation [55]. *BCL2* is one such anti-apoptotic gene [56]. It encodes the *Bcl-2* mitochondrial protein that allows cells to survive by inhibiting caspase activation by preventing cytochrome c release [57]. *Bcl-2* also protects cells from p53 and cMyc-induced apoptosis. This reduction in apoptosis is associated with a rise in *Bcl-2* levels in the AML and late myelodysplastic syndrome [58]. Thus, *Bcl-2* is an important target for therapy of several malignancies since it is a key player in cell proliferation imbalance.

Molecular docking results are present in Table 5 and Figure 8. The reference drug navitoclax showed the highest affinity for the receptor (−11.5 kcal/mol).

Affinities of the CSP extract components ranged between −7.8 kcal/mol (epicatechin gallate) to −5.7 kcal/mol (vanillic acid). The most active compounds were the catechins and rutin (−7.6 kcal/mol).

The docking results corroborated the cytotoxic activity observed in vitro as different molecules present in the CSP extract, especially the catechins and rutin, showed very good interaction affinity, the values of which were similar to those of the reference drugs and superior to the *ABL1* receptor. On the other hand, moderate docking affinities were noted for oleuropein (secoiridoid) and chlorogenic acid (phenolic acid), while weak affinities were obtained for vanillic acid (phenolic acid).

All molecules under investigation demonstrated high affinities for the receptor proteins. Among the CSP extract components, the epicatechin revealed the best affinity for the *ABL* kinase; epicatechin gallate was the most active for *ABL1* and *Bcl-2*, and catechin showed the highest affinity for the *FLT3*. Rutin exhibited excellent affinity for most of the receptors investigated. Therefore, our results suggest that antileukemic activity of the CSP extract arises from the synergistic anticancer action of the catechins and rutin molecules.

## 3. Materials and Methods

### 3.1. Chemicals and Reagents

The following commercial cell lines and reagents were used in the anticancer study: HL60 (ATCC ^®^ CCL240TM-Human acute promyelocytic leukemia), K562 (ATCC^®^ CCL-243TM–chronic myelogenous leukemia), and Vero cells (kidney epithetical cell line derived from an African Green Monkey). RPM 1640 and DMEM-Dulbecco’s Modified Eagle Medium both from Gibco (Rockville, MD, USA) were used as culture media. Heat inactivated fetal bovine serum with Penicillin, Amphotericin B and Streptomycin (FBS; Gibco, Dublin, Ireland) was used to supplement the media. The MTT, 3-(4,5-dimethyl thiazol-2-yl)-2, 5-diphenyl tetrazolium bromide was used in the cell viability assays (Merck, Darmstadt, Germany).

The following commercial solvent were used in the chromatographic analysis of the extract: acetonitrile and formic acid LC–MS grade purchased from Merck (Purity ≥ 99.9%).

The following commercial solvents were used during the preparation of the extract: hexane, methanol, *n*-butanol analytical grade (Merck, Purity ≥ 95 %)

### 3.2. Plant Material

*Coriandrum sativum* L. seeds were purchased from a local herbalist in July 2018 in the city of Fez, Morocco. They originated from northeastern Morocco in the region of Oujda (34°41′47.591″ lat; 1°57′2.13″ long.; altitude, 558 m) and were harvested one month before purchase. The seeds were identified and authenticated by Professor Amina Bari (a botanist at the USMBA) and a voucher specimen # BPRN28 was deposited in the Herbarium of the Laboratory of Biotechnology, Environment, Agrifood, and Health at the Faculty of Sciences Dhar el Mahraz of the USMBA, Fez, Morocco.

### 3.3. Preparation and Analysis of the Polyphenolic Extract

The seeds of *C. sativum* were thoroughly cleaned with distilled water and dried at room temperature. Subsequently, they were reduced to a coarse powder in an electric grinder (KRUPS; GX332850; Solingen, Germany), dried and then reduced to a fine powder. Since the powder contained a high amount of oil, the seeds were defatted with hexane (Merck, Purity ≥ 95 %) (3 × 30 mL for 10 g of powder) before extraction. The extraction was carried out in an ultrasound-assisted apparatus (HINOTEK; SB-100DT; Ningbo, China), 10 g of the defatted powder was mixed with methanol 70% and sonicated for 40 min at a frequency of 35 kHz. The liquid phase was separated by filtration and concentrated to dryness on a rotary evaporator (BUCHI; R-100 Rotavapor ^®^; New Castle, DE, USA; at 60 °C) to give a crude extract. The latter was mixed with the distilled water and extracted 2 times with *N*-butanol to get the polyphenol fraction [17]. The CSP extract was stored at 4 °C.

The CSP extract was analyzed by UHPLC/MS-MS on a Shimadzu Nexera XR LC 40 instrument, (Shimadzu Italia, Milan, Italy) equipped with a MS/MS detector (LCMS 8060, Shimadzu Italia) and controlled by Lab Solution software. The analysis was performed with the parameters detailed in [17] and the Supplementary Materials, using a flow injection on a Phenomenex Kinetex polar C18 column (3 × 100 mm^2^, 2.6 μm, Phenomenex, Torrance, CA, USA) and negative electrospray ionization (ESI-). The compounds were identified using an updated polyphenol in-house library taking into account the characteristic molecular fragment ions.

### 3.4. Cell Culture

The human cancer and Vero cell lines were obtained from the National Institute of Amazon Researches, Brazil. HL60 (ATCC ^®^ CCL-240TM, human histiocytic lymphoma) e K562 (ATCC ^®^ CCL-243TM, human chronic myelogenous leukemia) and Vero cell lines (2 × 10^4^ cells per well). were cultured into a 96-well plate containing 0.2 mL of RPMI medium (with 10% FBS, penicillin–streptomycin, and fungizone) per well, in an atmosphere of 5% CO_2_ at 37 °C for 24 h. After the formation of a sub-confluent monolayer, the cells were treated with different concentrations of the CSP extract (diluted in PBS with DMSO 0.5%) and incubated again at the same conditions for 24, 48, and 72 h.

### 3.5. Cytotoxicity Assay

We assessed the cytotoxicity of the CSP extract by the MTT assay. Sterile PBS and DMSO 0.5% were used as a negative control and DMSO 100% as a positive one. Subsequently, the medium was removed from all wells, and 10 µL of MTT (5 mg/mL in sterile PBS) diluted in 100 µL of DMEM medium (without phenol red to avoid misinterpretation) was added to the wells and incubated for 4 h at the same conditions as stated above. After that, the MTT was removed, and 50 µL of MTT lysis buffer was added to each well. The content was gently homogenized to dissolve the formazan crystals and then incubated for 10 min at 37 °C. Optical densities of the samples were measured on a microplate reader at a wavelength of 570 nm. The relative viability of the cells was estimated using the following equation:

$$Cell\;viability\;=\frac{A570\mathrm{of}\mathrm{the}\mathrm{treated}\mathrm{sample}}{A570\;\mathrm{of}\;\mathrm{the}\;\mathrm{untreated}\;\mathrm{sample}}\times 100$$

All tests were performed in triplicate.

### 3.6. Acute Toxicity Study

The toxicity test was conducted following the OECD 423 “dose adjustment” protocol [59] and consisted of testing the CSP extract at a dose of 2000 mg/kg bodyweight. The test was performed on 12 female Swiss albino mice and their behavior and number of deaths were checked twice a day for up to 14 days. After 15 h of fasting, they were divided into a control group consisting of 6 females receiving distilled water at a rate of 10 mL/kg and an experimental group consisting of 6 females receiving the CSP extract at a dose of 2000 mg/kg. After administration of the extract, the mice were continuously monitored within the first hour and then at 6 and 24 h after the treatment for any mortality or behavioral changes (agitation, lack of appetite, motor difficulties, or dyspnea). Those signs of toxicity were monitored daily for 14 days. On the 15th day, the number of dead animals was calculated, and the calculated values were transformed into a percentage, while the animals remaining alive were euthanized.

The mice were euthanized through anesthesia and blood was collected for hematological and biochemical analysis. The liver, kidney, and spleen were taken for organ control, rinsed in 0.9% saline, and weighed.

### 3.7. Molecular Docking

#### 3.7.1. Ligand Preparation

All chemical structures were retrieved as SDF 3D files from PubChem database (catechin CID: 9064; epicatechin CID: 72276; epicatechin gallate CID:107905; epigallocatechin CID:72277; gallocatechin CID:65084; oleuropein CID:5281544; rutin CID:5280805; chlorogenic acid: 1794427; vanillic acid CID:8486; nilotinib CID: 644241; danusetib CID: 11442891; quizartinib CID: 24889392) and then converted into a PDBQT format with AutoDockTools v1.5.6 [60] for the docking simulation. The Gasteiger partial charges were added, rotatable bonds were established, and non-polar hydrogen atoms were merged as part of the planning.

#### 3.7.2. Preparation of Receptors

The PDB file of each receptor was retrieved from the Protein Data Bank website (https://www.rcsb.org (accessed on 10 March 2021)) [61]. Precise X-ray crystal structures of the receptors were chosen based on their completeness, resolution, and fit with our objective. The selected receptors and their PIDs (Protein Data Bank identification codes) were as follows: *ABL* kinase (3vs9), *ABL1* (2v7a), *BCL2* (4lvt), *FLT3* (4rt7). The receptors were prepared by deleting water molecules, the default ligand, and heteroatoms using the Discovery Studio Visualizer v21 for windows [62]. AutoDockTools was used to open the updated receptors and apply polar hydrogen atoms and Gasteiger charges before converting them to the PDBQT format for further docking simulations.

#### 3.7.3. Docking Simulations

AutoDockTools was used to specify the grid box size for each receptor, and AutoDockVina [63] was used to run the docking simulations for different ligands and the four receptors. Exhaustiveness of the simulations was set to 24 to ensure that Vina would generate the maximum outcome in the shortest amount of time. Discovery Studio Visualizer v21 was used to generate images of the protein–ligand complexes.

### 3.8. Statistical Analysis

All data were represented as an arithmetic mean and a standard deviation (SD). Statistical analysis was carried out using Student’s *t*-test and ANOVA. A probability value of less than 0.05 was chosen as the criterion of statistical significance. *: *p* < 0.05; **: *p* < 0.01; ***: *p* < 0.001 when compared to negative control (untreated cells). IC_50_ was calculated by a non-linear regression.

## 4. Conclusions

The paper represents the first data on of the effects of the polyphenolic extract of *C. sativum* seeds on two leukemic cell lines (K562 and HL-60). Our results demonstrated a dose-dependent cytotoxicity against the tumor cells and no effect on the viability and growth of a normal cell line (Vero). No signs of toxicity during the in vivo acute toxicity study demonstrated that CSP ais a very safe and effective combination of biologically active molecules.

All CSP components interacted positively with the *ABL* kinase, *ABL1*, *BCL2*, and *FLT3* receptors in the tumor cells, and thus may explain a possible mode of action of the extract. Taking into a consideration all the data obtained in the in vitro and in vivo experiments together with in silico results, the antileukemic activity of the CSP extract could be mainly attributed to a synergic combination of the catechins and rutin. The extract may thus be proposed as a phytomedical alternative to conventional treatments and chemotherapy, owing to its powerful activity and lack of side effects.

Our findings demonstrated that catechins directly associate with cellular targets, e.g., the cell surface receptors. However, more research using isolated molecules of the CSP extract is needed to further characterize its anticancer activity and its mechanism of action.

## Figures and Tables

**Table 1:** IC_50_ of the CSP extract, its components, and the reference molecules towards K562, HL60, and Vero cell lines.

Compounds	IC_50_ (µM)		
	K562 Cell Line	HL60 Cell Line	Vero Cell Line
CSP	16.86	11.75	>100
Nilotinib [36]	0.0243	>2	-
Danusertib [37]	0.15	3.06	-
Quizartinib [38]	>10	>10	-
Navitoclax [39]	0.4	-	-
Catechin [40]	-	>100	-
Epicatechin [40]	-	>100	-
Epigallocatechin [41]	-	107.7	-
Epigallocatechin Gallate [41]	-	60	-
Rutin [41,42]	-	14	-
Vanillic acid [43]	56	-	-

**Table 2:** Assessment of a relative weight of organs in the acute toxicity study.

Groups	Liver (g) *	Kidney (g) *	Spleen (g) *
Control	8.65 ± 0.52	1.79 ± 0.18	0.74 ± 0.15
CSP, 2000 mg/kg	7.96 ± 0.05	1.78 ± 0.02	0.75 ± 0.05

* Data are presented as mean ± SD (*n* = 3).

**Table 3:** Biochemical parameters of mice.

Parameter	Control *	CSP 2 g/kg *
Urea (g/L)	0.28 ± 0.02	0.21 ± 0.03
Creatinine (mg/L)	3.40 ± 0.31	3.80 ± 0.25
ALT (U/L)	45.80 ± 2.11	58.20 ± 4.71
AST (U/L)	397.7 ± 30.37	354.7 ± 23.42
Triglycerides (mg/dL)	87.33 ± 16.25	85.66 ± 12.85
Total Cholesterol (mg/dL)	93.00 ± 7.93	91.33 ± 8.96
HDL (mg/dL)	45.33 ± 3.05	41.33 ± 4.72
VLDL (mg/dL)	17.33 ± 3.51	17.00 ± 2.64
Total Proteins (gm/dL)	6.16 ± 0.15	5.90 ± 0.17
Albumin (gm/dL)	3.33 ± 0.15	3.06 ± 0.30
ALP (IU/L)	92.33 ± 9.29	78.66 ± 4.50
Total Bilirubin (mg/dL)	1.00 ± 0.26	0.96 ± 0.05
Direct Bilirubin (mg/dL)	0.40 ± 0.10	0.43 ± 0.15

* Data were presented as mean ± SD (*n* = 3).

**Table 4:** Hematological parameters of mice.

Parameter.	Control *	CSP 2 g/kg *
Total Hb (g/dL)	12.60 ± 1.15	12.96 ± 0.85
Total RBC (106/µL)	11.20 ± 0.35	10.83 ± 0.22
Total WBC (103/µL)	2.22 ± 0.85	3.00 ± 1.12
Platelet Count (103/µL)	512.33 ± 37.68	502.0 ± 45.51
HCT (%)	47.20 ± 3.27	43.10 ± 1.71
Granulocytes (%)	22.76 ± 2.01	20.06 ± 2.36
Lymphocytes (%)	55.26 ± 4.73	63.36 ± 6.29
Monocytes (%)	13.16 ± 1.76	12.62 ± 2.58

* Data are presented as mean ± SD (*n* = 3).

**Table 5:** Docking results for different ligands and reference molecules with the receptors.

Receptor	Reference	Affinity (kcal/mol)								
		CAT	EPI	EPG	EGC	GC	OLE	RU	CA	VA
ABL kinase	−9.9 ^a^	−8.8	−9.6	−8.1	−8.2	−7.2	−6.4	−9.3	−7.6	−6.5
ABL1	−8.5 ^b^	−8.2	−7.7	−9.2	−7.9	−7.9	−6.9	−8.6	−6.8	−5.4
BCL2	−11.5 ^c^	−6.8	−6.8	−7.8	−6.9	−6.8	−6.8	−7.6	−6.9	−5.3
FLT3	−10.1 ^d^	−9.1	−8.3	None	−8.3	−8.9	None	None	−8.3	−5.7

CAT: catechin; EPI: epicatechin; EGC: epigallocatechin; GC: gallocatechin; OLE: oleuropein; RU: rutin; CA: chlorogenic acid and VA: vanillic acid; a: nilotinib b: danusertib c: quizartinib d: navitoclax.

## Data Availability

All data is available within the article and Supplementary Material.

## Supplementary Materials

The following are available online at https://www.mdpi.com/article/10.3390/ph14080770/s1, Figure S1: Molecular peaks of the CSP extract and their subsequent fragmentation in MS/MS experiment, Table S1: Identified fragments of the CSP extract.

## References

[1] Blumenreich M.S., Walker H.K., Hall W.D., Hurst J.W. (1990). The white blood cell and differential count. Clinical Methods: The History, Physical, and Laboratory Examinations.

[2] Siegel R.L., Miller K.D., Jemal A. (2020). Cancer Statistics, 2020. CA.

[3] Bhat A.A., Younes S.N., Raza S.S., Zarif L., Nisar S., Ahmed I., Mir R., Kumar S., Sharawat S.K., Hashem S. (2020). Role of non-coding RNA networks in leukemia progression, metastasis and drug resistance. Mol. Cancer.

[4] Matsukawa T., Aplan P.D. (2020). Clinical and molecular consequences of fusion genes in myeloid malignancies. Stem Cells.

[5] Shankar D.B., Sakamoto K.M. (2004). The role of cyclic-amp binding protein (CREB) in leukemia cell proliferation and acute leukemias. Leuk. Lymphoma.

[6] Ikeda A., Shankar D.B., Watanabe M., Tamanoi F., Moore T.B., Sakamoto K.M. (2006). Molecular targets and the treatment of myeloid leukemia. Mol. Genet. Metab..

[7] Corey S.J. (2005). New agents in the treatment of childhood leukemias and myelodysplastic syndromes. Curr. Oncol. Rep..

[8] Ling Y., Xie Q., Zhang Z., Zhang H. (2018). Protein kinase inhibitors for acute leukemia. Biomark. Res..

[9] Saraswathy M., Gong S. (2013). Different strategies to overcome multidrug resistance in cancer. Biotechnol. Adv..

[10] Roy A., Jauhari N., Bharadvaja N. (2018). Medicinal plants as a potential source of chemopreventive agents. Anticancer Plants: Natural Products and Biotechnological Implements.

[11] Mechchate H., Es-safi I., Haddad H., Bekkari H., Grafov A., Bousta D. (2020). Combination of catechin, epicatechin, and rutin:optimization of a novel complete antidiabetic formulation using a mixture design approach. J. Nutr. Biochem..

[12] Mundlia J., Ahuja M., Kumar P. (2020). Enhanced biological activity of polyphenols on conjugation with gellan gum. Int. J. Polym. Mater. Polym. Biomater..

[13] Szwajgier D., Paduch R., Kukuła-Koch W., Polak-Berecka M., Waśko A. (2020). Study on biological activity of bread enriched with natural polyphenols in terms of growth inhibition of tumor intestine cells. J. Med. Food.

[14] Nadeem M., Muhammad Anjum F., Issa Khan M., Tehseen S., El-Ghorab A., Iqbal Sultan J. (2013). Nutritional and medicinal aspects of coriander (*Coriandrum Sativum* L.): A review. Br. Food J..

[15] Jana S., Patra K., Sarkar S., Jana J., Mukherjee G., Bhattacharjee S., Mandal D.P. (2014). Antitumorigenic potential of linalool is accompanied by modulation of oxidative stress: An in vivo study in sarcoma-180 solid tumor model. Nutr. Cancer.

[16] Villaseñor I.M., Bravo N.F.C., Ortega K.J.L. (2009). Anti-skin tumor activity of nutraceuticals from strawberry, coriander, red coral lettuce and chinese chives. Philipp. Agric. Sci..

[17] Mechchate H., Es-safi I., Amaghnouje A., Boukhira S., Alotaibi A.A., Al-zharani M., Nasr F.A., Noman O.M., Conte R., Amal E.H.E.Y. (2021). Antioxidant, anti-inflammatory and antidiabetic proprieties of LC-MS/MS identified polyphenols from coriander seeds. Molecules.

[18] Bernatoniene J., Kopustinskiene D.M. (2018). The role of catechins in cellular responses to oxidative stress. Molecules.

[19] Fernando W., Rupasinghe H.P.V., Hoskin D.W. (2019). Dietary phytochemicals with anti-oxidant and pro-oxidant activities: A double-edged sword in relation to adjuvant chemotherapy and radiotherapy?. Cancer Lett..

[20] Naghma K., Hasan M. (2013). Tea and health: Studies in humans. Curr. Pharm. Des..

[21] Zhang L., Ho C.-T., Zhou J., Santos J.S., Armstrong L., Granato D. (2019). Chemistry and biological activities of processed camellia sinensis teas: A comprehensive review. Compr. Rev. Food Sci. Food Saf..

[22] Guo Y., Zhi F., Chen P., Zhao K., Xiang H., Mao Q., Wang X., Zhang X. (2017). Green tea and the risk of prostate cancer: A systematic review and meta-analysis. Medicine.

[23] Najaf Najafi M., Salehi M., Ghazanfarpour M., Hoseini Z.S., Khadem-Rezaiyan M. (2018). The association between green tea consumption and breast cancer risk: A systematic review and meta-analysis. Phytother. Res..

[24] Naponelli V., Ramazzina I., Lenzi C., Bettuzzi S., Rizzi F. (2017). Green tea catechins for prostate cancer prevention: Present achievements and future challenges. Antioxidants.

[25] Rashmi H.B., Negi P.S. (2020). Phenolic acids from vegetables: A review on processing stability and health benefits. Food Res. Int..

[26] Anantharaju P.G., Gowda P.C., Vimalambike M.G., Madhunapantula S.V. (2016). An overview on the role of dietary phenolics for the treatment of cancers. Nutr. J..

[27] Kumar N., Goel N. (2019). Phenolic acids: Natural versatile molecules with promising therapeutic applications. Biotechnol. Rep..

[28] Santana-Gálvez J., Castrejón J.V., Serna-Saldívar S.O., Jacobo-Velázquez D.A. (2020). Anticancer potential of dihydrocaffeic acid: A chlorogenic acid metabolite. CyTA J. Food.

[29] Matsunaga K., Katayama M., Sakata K., Kuno T., Yoshida K., Yamada Y., Hirose Y., Yoshimi N., Mori H. (2002). Inhibitory effects of chlorogenic acid on azoxymethane-induced colon carcinogenesis in male F344 rats. Asian Pac. J. Cancer Prev..

[30] Gong J., Zhou S., Yang S. (2019). Vanillic acid suppresses HIF-1α expression via inhibition of MTOR/P70S6K/4E-BP1 and Raf/MEK/ERK pathways in human colon cancer HCT116 cells. Int. J. Mol. Sci..

[31] Nouri Z., Fakhri S., Nouri K., Wallace C.E., Farzaei M.H., Bishayee A. (2020). Targeting multiple signaling pathways in cancer: The rutin therapeutic approach. Cancers.

[32] Caparica R., Júlio A., Araújo M.E.M., Baby A.R., Fonte P., Costa J.G., de Almeida T.S. (2020). Anticancer activity of rutin and its combination with ionic liquids on renal cells. Biomolecules.

[33] Chen H., Miao Q., Geng M., Liu J., Hu Y., Tian L., Pan J., Yang Y. (2013). Anti-tumor effect of rutin on human neuroblastoma cell lines through inducing G2/M cell cycle arrest and promoting apoptosis. Sci. World J..

[34] Grzesik M., Naparło K., Bartosz G., Sadowska-Bartosz I. (2018). Antioxidant properties of catechins: Comparison with other antioxidants. Food Chem..

[35] Shirakami Y., Shimizu M. (2018). Possible mechanisms of green tea and its constituents against cancer. Molecules.

[36] Deguchi Y., Kimura S., Ashihara E., Niwa T., Hodohara K., Fujiyama Y., Maekawa T. (2008). Comparison of imatinib, dasatinib, nilotinib and INNO-406 in imatinib-resistant cell lines. Leuk. Res..

[37] Gontarewicz A., Balabanov S., Keller G., Colombo R., Graziano A., Pesenti E., Benten D., Bokemeyer C., Fiedler W., Moll J. (2008). Simultaneous targeting of aurora kinases and Bcr-Abl kinase by the small molecule inhibitor PHA-739358 is effective against imatinib-resistant BCR-ABL mutations including T315I. Blood.

[38] Kampa-Schittenhelm K.M., Heinrich M.C., Akmut F., Döhner H., Döhner K., Schittenhelm M.M. (2013). Quizartinib (AC220) is a potent second generation class III tyrosine kinase inhibitor that displays a distinct inhibition profile against mutant-FLT3, -PDGFRA and -KIT isoforms. Mol. Cancer.

[39] Casson L., Howell L., Mathews L.A., Ferrer M., Southall N., Guha R., Keller J.M., Thomas C., Siskind L.J., Beverly L.J. (2013). Inhibition of ceramide metabolism sensitizes human leukemia cells to inhibition of BCL2-like proteins. PLoS ONE..

[40] Pan X., Matsumoto M., Nishimoto Y., Ogihara E., Zhang J., Ukiya M., Tokuda H., Koike K., Akihisa M., Akihisa T. (2014). Cytotoxic and nitric oxide production-inhibitory activities of limonoids and other compounds from the leaves and bark of melia azedarach. Chem. Biodivers..

[41] Han D.H., Kim J.H. (2009). Difference in growth suppression and apoptosis induction of EGCG and EGC on human promyelocytic leukemia HL-60 cells. Arch. Pharm. Res..

[42] Araújo K.C.F., de MBCosta E.M., Pazini F., Valadares M.C., de Oliveira V. (2013). Bioconversion of quercetin and rutin and the cytotoxicity activities of the transformed products. Food Chem. Toxicol..

[43] Chiang L.-C., Chiang W., Chang M.-Y., Ng L.-T., Lin C.-C. (2003). Antileukemic activity of selected natural products in taiwan. Am. J. Chin. Med..

[44] Kharchoufa L., Bouhrim M., Bencheikh N., Addi M., Hano C., Mechchate H., Elachouri M. (2021). Potential Toxicity of Medicinal Plants Inventoried in Northeastern Morocco: An Ethnobotanical Approach. Plants.

[45] Bolkent S., Yanardag R., Ozsoy-Sacan O., Karabulut-Bulan O. (2004). Effects of parsley (*Petroselinum crispum*) on the liver of diabetic rats: A morphological and biochemical study. Phytother. Res..

[46] Lorusso D., Bria E., Costantini A., Di Maio M., Rosti G., Mancuso A. (2017). Patients’ perception of chemotherapy side effects: Expectations, doctor–patient communication and impact on quality of life—An italian survey. Eur. J. Cancer Care.

[47] Haq M.I., Nixon J., Stanley A.J. (2018). Imatinib and liver toxicity. BMJ Case Rep..

[48] Efficace F., Baccarani M., Breccia M., Alimena G., Rosti G., Cottone F., Deliliers G.L., Baratè C., Rossi A.R., Fioritoni G. (2013). Chronic fatigue is the most important factor limiting health-related quality of life of chronic myeloid leukemia patients treated with imatinib. Leukemia.

[49] Hensley M.L., Ford J.M. (2003). Imatinib treatment: Specific issues related to safety, fertility, and pregnancy. Semin. Hematol..

[50] Schirrmacher V. (2019). From chemotherapy to biological therapy: A review of novel concepts to reduce the side effects of systemic cancer treatment. Int. J. Oncol..

[51] Iacobucci I. (2008). Mechanism of resistance to tyrosine kinase inhibitors in philadelphia-positive acute lymphblastic leukaemia (all): From genetic alterations to impaired RNA editing. Mol. Sci..

[52] Park H., Hong S., Kim J., Hong S. (2013). Discovery of picomolar abl kinase inhibitors equipotent for wild type and T315I mutant via structure-based de novo design. J. Am. Chem. Soc..

[53] Kazi J.U., Rönnstrand L. (2019). The role of src family kinases in FLT3 signaling. Int. J. Biochem. Cell Biol..

[54] Gruszka A.M., Valli D., Alcalay M. (2019). Wnt signalling in acute myeloid leukaemia. Cells.

[55] Shokouhian M., Bagheri M., Poopak B., Chegeni R., Davari N., Saki N. (2020). Altering chromatin methylation patterns and the transcriptional network involved in regulation of hematopoietic stem cell fate. J. Cell. Physiol..

[56] Distelhorst C.W., Bootman M.D. (2019). Creating a new cancer therapeutic agent by targeting the interaction between Bcl-2 and IP3 receptors. Cold Spring Harb. Perspect. Biol..

[57] Santucci R., Sinibaldi F., Cozza P., Polticelli F., Fiorucci L. (2019). Cytochrome c: An extreme multifunctional protein with a key role in cell fate. Int. J. Biol. Macromol..

[58] Yogarajah M., Stone R.M. (2018). A concise review of BCL-2 inhibition in acute myeloid leukemia. Expert Rev. Hematol..

[59] OECD (2002). Test. No. 423: Acute Oral toxicity—Acute Toxic Class. Method. OECD Guidelines for the Testing of Chemicals, Section 4.

[60] Morris G.M., Huey R., Lindstrom W., Sanner M.F., Belew R.K., Goodsell D.S., Olson A.J. (2009). Autodock4 and autodocktools4: Automated docking with selective receptor flexibility. J. Comput. Chem..

[61] Berman H.M., Battistuz T., Bhat T.N., Bluhm W.F., Bourne P.E., Burkhardt K., Feng Z., Gilliland G.L., Iype L., Jain S. (2002). The protein data bank. Acta Crystallogr. Sect. D Biol. Crystallogr..

[62] (2020). Discovery Studio Visualizer Version 21.

[63] Trott O., Olson A.J. (2009). Autodock vina: Improving the speed and accuracy of docking with a new scoring function, efficient optimization, and multithreading. J. Comput. Chem..

