# Supplementary material for: Antileukemic Activity and Molecular Docking Study of a Polyphenolic Extract from Coriander Seeds
Source: Pharmaceuticals (Basel). 2021 Aug 5;14(8):770. doi: 10.3390/ph14080770 (PMC8400422; doi:10.3390/ph14080770)
Supplement: Supplementary file 1 [file pharmaceuticals-14-00770-s001.zip › pharmaceuticals-1272577-supplementary.pdf]

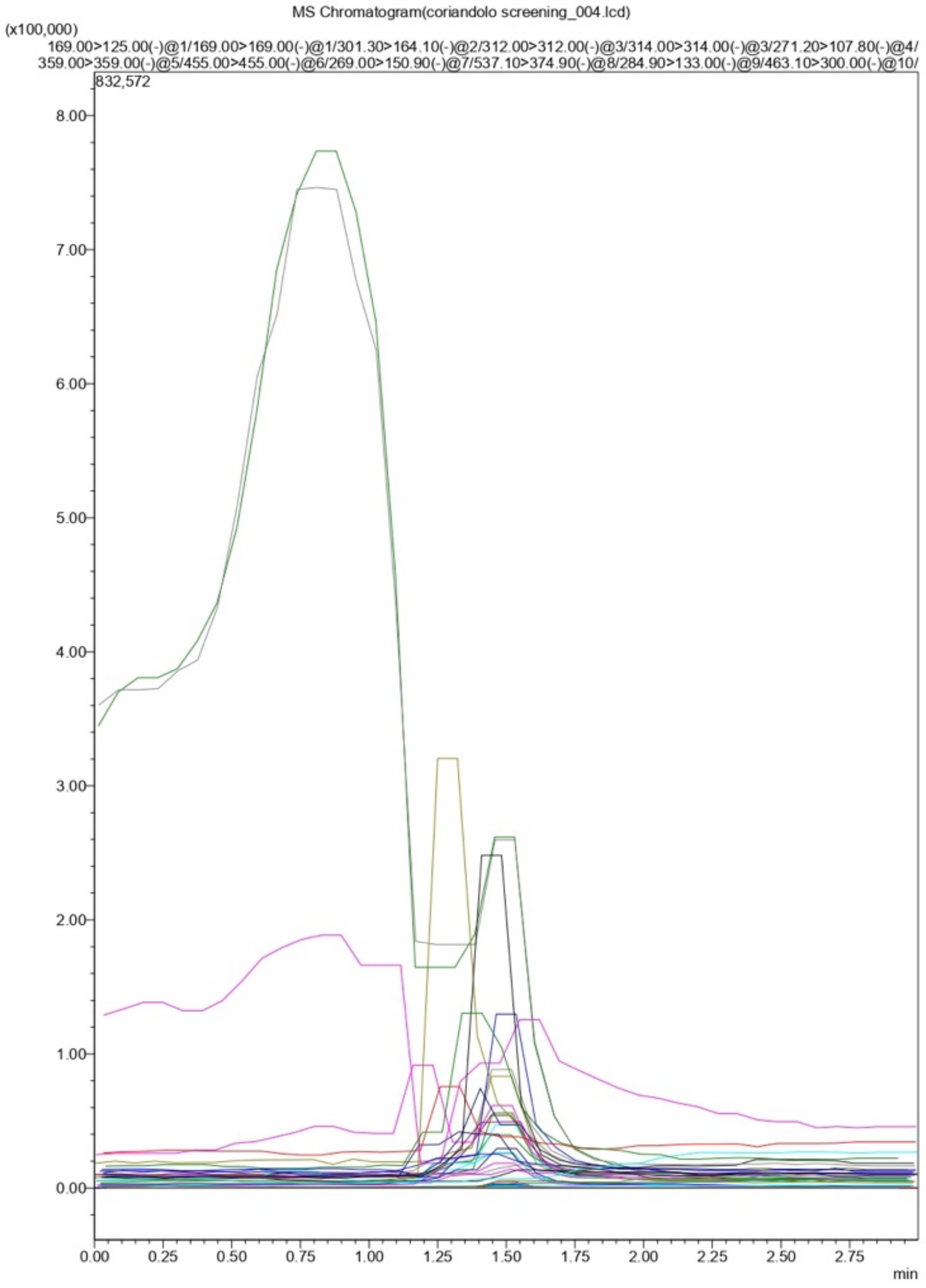

Figure S1: Molecular peaks of the CSP extract and their subsequent fragmentation in MS/MS experiment

Table S1: Identified fragments of the CSP extract

Molecule	Identified fragments	*Coriandrium sativum* seeds (PCS)	Standard in ACN (dil 1:1000)
Vanillic acid	167.00>123.00<br>167.00>167.00	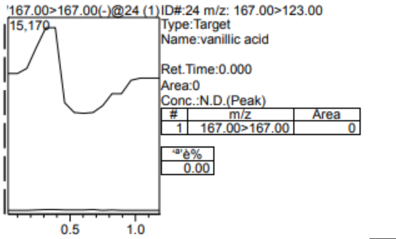	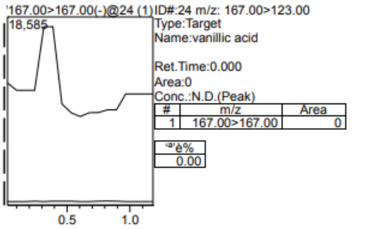
Chlorogenic acid	353.00>190.00<br>353.00>353.00	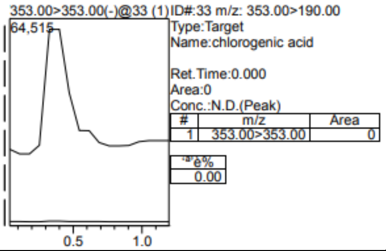	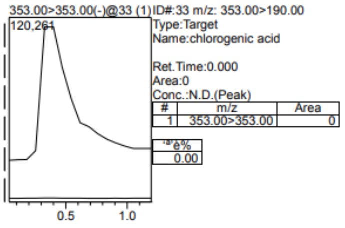
Oleuropein	539.00>539.00	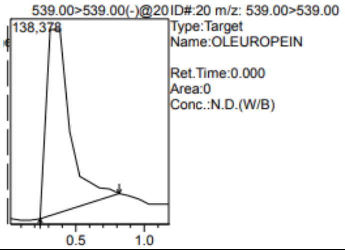	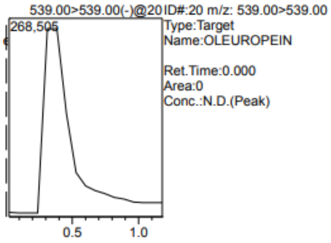
Epicatechin gallate	441.00>289.00<br>441.00>441.00	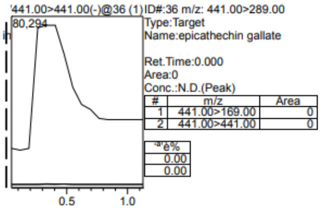	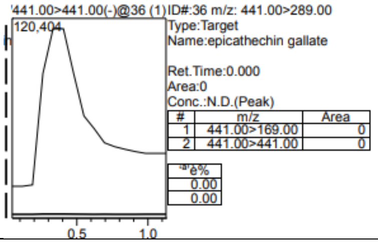
Catechin\ Epicatechin	289.00>245.00<br>289.00>289.00	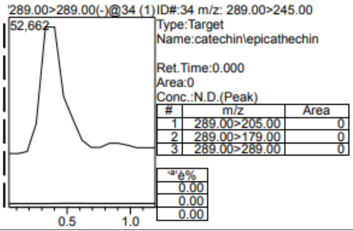	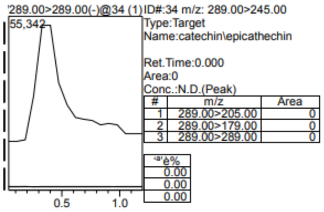
Gallocatechin\ Epigallocatechin	457.00>169.00<br>457.00>457.00	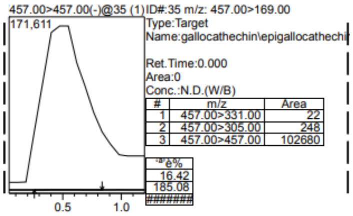	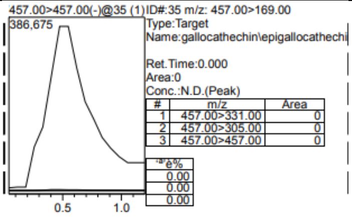
Rutin	609.00>301.00<br>609.00>609.00	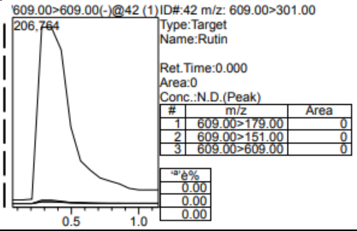	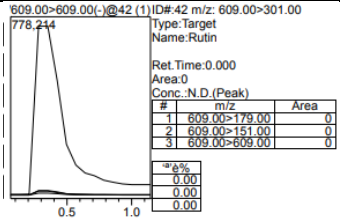
